# Damaging the Integrated HIV Proviral DNA with TALENs

**DOI:** 10.1371/journal.pone.0125652

**Published:** 2015-05-06

**Authors:** Christy L. Strong, Horacio P. Guerra, Kiran R. Mathew, Nervik Roy, Lacy R. Simpson, Martin R. Schiller

**Affiliations:** Nevada Institute of Personalized Medicine and School of Life Sciences, University of Nevada Las Vegas, Las Vegas, NV, United States of America; QIMR Berghofer Medical Research Institute, AUSTRALIA

## Abstract

HIV-1 integrates its proviral DNA genome into the host genome, presenting barriers for virus eradication. Several new gene-editing technologies have emerged that could potentially be used to damage integrated proviral DNA. In this study, we use transcription activator-like effector nucleases (TALENs) to target a highly conserved sequence in the transactivation response element (TAR) of the HIV-1 proviral DNA. We demonstrated that TALENs cleave a DNA template with the HIV-1 proviral target site *in vitro*. A GFP reporter, under control of HIV-1 TAR, was efficiently inactivated by mutations introduced by transfection of TALEN plasmids. When infected cells containing the full-length integrated HIV-1 proviral DNA were transfected with TALENs, the TAR region accumulated indels. When one of these mutants was tested, the mutated HIV-1 proviral DNA was incapable of producing detectable Gag expression. TALEN variants engineered for degenerate recognition of select nucleotide positions also cleaved proviral DNA *in vitro* and the full-length integrated proviral DNA genome in living cells. These results suggest a possible design strategy for the therapeutic considerations of incomplete target sequence conservation and acquired resistance mutations. We have established a new strategy for damaging integrated HIV proviral DNA that may have future potential for HIV-1 proviral DNA eradication.

## Introduction

Human Immunodeficiency Virus (HIV), the causative agent of Acquired Immunodeficiency Syndrome (AIDS), is a pathogenic retrovirus that integrates a proviral DNA copy of its genome into the genome of host cells. Three decades of research and development have produced many antiretroviral (ARV) drugs that, when combined in Highly Active Antiretroviral Therapy (HAART) can reduce the plasma viral load in infected patients, and even shut down viral production [1]. But even with chronic HAART treatment, an integrated copy of proviral HIV DNA remains in latent cells, which can re-establish viral production and cause a rebound, producing plasma viremia [2].

The persistent latent HIV reservoir is a major barrier to HIV treatment [3]. The most prominent current strategy to address HIV latency is, while under HAART therapy, to reactivate latently infected cells so that they can be targeted by the immune system [2,4–6]. A major problem with this approach is that specific reactivation of only latent cells has not been achieved and nonspecific reactivation of T-cells can lead to a cytokine storm [7]. Furthermore, even replacing the immune system does not guarantee a cure.

An approach to eradicate or damage the integrated HIV proviral DNA is needed. One promising new approach is genome editing with engineered nucleases (GEEN). There are four main technologies used for GEEN: (1) meganucleases; (2) zinc finger nucleases (ZFN); (3) transcription activator-like effector nucleases (TALENs); and (4) clustered regulatory interspaced short palindromic repeat (CRISPR)/Cas-based RNA-guided DNA endonucleases. These technologies catalyze double strand breaks in genomic DNA that are thought to be repaired in cells by endogenous nonhomologous end joining (NHEJ). These repairs often produce mistake insertions or deletions, introducing indels into the targeted DNA, thus mutating the genomic DNA.

Others have tested Tre recombinase, zinc finger nucleases, and CRISPR/Cas-9 in attempts to target the integrated HIV-1 proviral DNA in cells [8–13]. One potential limitation of these GEEN approaches is that the HIV-1 proviral DNA displays few long stretches with highly conserved nucleotides, thus GEEN treatment may be prone to HIV-1 escape mutations.

We explored using the TALEN based technology to mutate and thus inactivate the HIV-1 proviral DNA because TALENs are the only GEEN where the targeting construct can encode specific degeneracy for the DNA recognition site, thus can be engineered to potentially inhibit escape mutations [14]. TALENs also are reported to have very high damage efficiencies of >50% achieved in several systems [15–20]. TALENs have flexibility in the target sequences, whereas meganucleases and ZFNs have a more limited breadth [21–24]. TALENs have high specificity achieved in some systems evaluated by exome sequencing with limited off-target editing and toxicity [25,26]. ZFNs have reported off-target editing sites, as well as CRISPR/Cas where sites with multiple base pairs that differ from the guide RNA can be edited [23,27,28]. The use of TALENs for treating HIV latency has been recently suggested [29,30]. Further support for using this approach to treat HIV come from a recent report where TALENs were effectively used to disable the episomal Hepatitis B Virus (HBV) genome and reduce viral load in cells and animals [31,32].

Herein, we have engineered a custom TALEN pair of HIV Targeted-TALENs (HT-TALENs) to specifically target a highly conserved region of the HIV-1 genome. We also built and tested a NS-TALEN designed with some degenerate recognition to accommodate escape mutations in regions where viral genome mutations have been previously observed. We report that both TALEN pairs can be used to damage the integrated HIV-1 proviral DNA in cultured cells infected with HIV-1. To our knowledge, this is the first demonstration that the full-length integrated HIV-1 proviral DNA can be mutated and protein expression negatively affected by introduction of TALENs, and thus inactivated in cells. This suggests a new promising alternative approach for treating viral latency.

## Materials and Methods

### Bioinformatics analysis of HIV-1 genome

HIV-1 Sub-type B DNA sequences for the complete genome and the 5’LTR, 5’LTR(R), 5’LTR(U3), 5’LTR(U5), GAGPOL, RRE, RT, TAR, ENV regions of the genome were downloaded from the Los Alamos HIV Sequence Database (http://www.hiv.lanl.gov/) and converted into comma-delimitated files using a custom script. The files were then loaded, aligned with ClustalΩ [33], and positional conservation was calculated with Microsoft Excel. Regions with stretches of bases that held the most positional conservation were selected as potential target regions. The strongest target region, encompassing TAR, was obtained from analysis of the 226 sequences encompassing the HIVB5LTR. The 5’ HT-TALEN and 5’ NS-TALEN binding sites encompass nucleotide positions 459–478 (HIV-1 HXB2 accession number K03455), while the 3’ HT-TALEN binding site encompasses nucleotide positions 499–515 (HIV-1 HXB2 accession number K03455).

### Design and construction of TALEN plasmids

A FASTA file for the HIV-1 Sub-type B 5’LTR HXB2 DNA sequence (accession number K03455) was input into the ZiFiT Webtool (http://zifit.partners.org/ZiFiT/) to retrieve a schematic for building TALEN constructs using the REAL Assembly Kit [34–36]. Plasmid DNA constructs for the HT-TALENs were built using the Joung Lab REAL Assembly TALEN kit (AddGene), following the REAL Assembly method as described [36]. Identity of correct HT-TALEN DNA clones was confirmed by sequence analysis (Beckman Coulter Genomics).

### *In vitro* transcription/translation of HT- and NS-TALENs and cleavage reactions

The target template DNA to be used in cleavage reactions was synthesized by Polymerase Chain Reaction (PCR) (HotStarTaq Plus Master Mix, Qiagen) using forward primer U3BamHI75F (CAGCTGGATCCTGATTGGCAG) and reverse primer GagSalI804Rev (GGGTGCGAGAGCGTCGACGACGG) to amplify a 747 bp product from plai.2 proviral DNA[37](NIH AIDS Reagent Program, catalog no. 2532). To generate a mutant target template, overlap extension of two PCR products was performed, followed by a PCR using a forward primer (U3BamHI75F) and a reverse primer (GagSalI804Rev). PCR product 1 (520 bp) was generated using plai.2 (a full-length HIV proviral DNA) as a template, U3BamHIFor and a randomized reverse primer (Random5’siteRev: CAGGCTCNNATCTGGTCNNNCNA). PCR product 2 (355 bp) was generated using plai.2 as a template, a randomized forward primer (Random5’siteFor: CTCTNGNNNGACCAGATNNGAGC), and GagSalI804Rev. The generated insert was ligated into SalI/BamHI digested pGEX6P3 (GE Healthcare Sciences).

*In vitro* transcription/translation reactions were performed using the TnT Quick Coupled Transcription/Translation System (Promega). Reactions consisted of 500 ng of each HT-TALEN pair DNA plasmid, 20 μL of TNT T7 Quick Master Mix, 0.5 μL Methionine (1 mM), 500ng target template DNA, and 2.5 μL H20. The reactions were incubated at 30°C for 2 hours. Aliquots were analyzed by Western blot and to the remaining reaction (20μL) was added to 100 μL of cleavage reaction buffer [18,38]. The samples were then incubated for an additional 3 hours at 30°C followed by Rnase A (20 μg) treatment for 15 minutes. DNA from the samples was purified (Wizard SV Gel and PCR Clean-Up System,) and ethanol precipitated to concentrate the samples. Concentrated samples were then run on a 1% 1XTAE agarose gel to visualize the target template and cleaved product DNAs. Image J software was used to quantify bands to determine cleavage efficiency [39]. These experiments were repeated 2–3 times.

### Cell culture and transfection

HeLa-tat-III/LTR/d1EGFP cells [40] were maintained in Dulbecco's modified Eagle's medium (DMEM) supplemented with 10% Fetal Bovine Serum (Fisher Scientific), 1% penicillin and streptomycin (Sigma) and 1mg/mL G418 (Fisher Scientific). HeLa/LAV cells [41,42] and pEAK Rapid cells (derived from HEK293 cells, Edge Biosystems) were maintained in Dulbecco's modified Eagle's medium (DMEM) supplemented with 10% Fetal Bovine Serum (Fisher Scientific), and 1% penicillin and streptomycin (Sigma). Transient transfections of both HeLa-tat-III/LTR/d1EGFP and HeLa/LAV cells was performed using the Trans-IT HeLa-MONSTER transfection kit (Mirus). Transient transfection of pEAK Rapid cells was performed using the Trans-IT 2020 transfection kit (Mirus). Cells were harvested 48 hours post-transfection.

### Flow cytometry

Cytotoxicity was determined for transiently transfected HeLa/LAV (pRSET.mCherry expression vector, HT-TALEN pair, NS-TALEN pair) samples in addition to control samples. Samples were harvested for Annexin V staining 72 hours post-transfection (FITC Annexin V Apoptosis Detection Kit, BD). Each sample-type was performed in triplicate. Wells were trypsinized (0.25% Trypsin), resuspended in 1 mL of phosphate buffered saline (PBS), and then centrifuged at 156 x g for 5 minutes. Samples were then gently resuspended in 1 mL HEPES buffer and centrifuged at 156 x g. for 5 minutes. Samples were gently resuspended in 50 μL HEPES buffer and 3 μL Annexin V was added to each sample, excluding the negative controls. Samples were incubated on ice for 20 minutes in the dark. Samples were centrifuged at 156 x g for 5 minutes, followed by a 1mL ice cold HEPES buffer wash. Samples were resuspended in a 4% paraformaldehyde solution and incubated at room temperature in the dark for 3 hours. Samples were centrifuged at 156 x g for 5 minutes. Samples were then washed in 1 mL PBS and then gently resuspended in 300μL PBS to prepare them for flow analysis.

TALEN efficiency was determined by number of mCherry/Green Fluorescent Protein (GFP) vs. mCherry-only positive cells recorded 72 hours post-transfection in transiently transfected HeLa-tat-III/LTR/d1EGFP cells (pRSET.mCherry expression vector, co-transfected HT-TALEN pair and pRSET.mCherry expression vector, cotransfected NS-TALEN pair and pRSET.mCherry expression vector). Each sample-type was performed in triplicate. Wells were trypsinized (0.25% Trypsin), resuspended in 1 mL PBS, and then centrifuged at 156 x g for 5 minutes. Samples were fixed in 4% paraformaldehyde, washed once with PBS and then resuspended in 500 μL PBS prior to flow analysis.

Flow cytometry data was acquired using a FACSCalibur Flow cytometer (Becton Dickinson). The blue laser (488nm) was used for detecting GFP while the red laser (635nm) was used for mCherry. 10,000 events were acquired for each sample. Flow cytometry analysis was performed using FlowJo (Tree Star) software. Non-fluorescent samples were used to determine thresholds. mCherry-positive only samples and GFP-positive only samples were used to set gating thresholds. Dose-response curves were generated by counting cells using different mCherry thresholds. Statistical analysis for cytotoxicity experiments was performed using Analysis of Variance (ANOVA) and statistical differences in slopes from TALEN dose-response curves were determined with a one-tailed t-test.

### Protein analysis

Cells were washed and lysed in PBS. One-half of the cell lysate was used for genomic DNA purification (see below), while the other half was combined with 2X Sodium Dodecyl sulfate (SDS) protein buffer for protein analysis. The protein samples were freeze/thawed three times, boiled at 95°C for 5 minutes, then loaded onto a 4–12% Bis-tris protein gel (Nupage, Life Technologies). Proteins in the gel were transferred onto a PVDF membrane (Immobilon-P, Millipore), blocked with 5% milk/PBS, and then probed with select primary antibodies. Primary antibodies used included: mouse anti-actin, mouse anti-Flag-HRP conjugate (SLBD 9930, Sigma Aldrich), mouse anti-capsid [43], and rabbit anti-Flag (A1113, Santa Cruz). Secondary antibodies used included: Goat anti-rabbit HRP and Rabbit anti-mouse HRP conjugates (GE Life Sciences). Proteins were visualized using chemiluminescence (Super Signal West Pico Chemiluminescent Substrate, Thermo Scientific) on an Automated Biospectrum Imaging System (UVP). All Western analyses were repeated 2–3 times

### Genomic DNA analysis

Genomic DNA was purified from cell lysates using a PureLink Genomic DNA kit (Life Technologies). PCRs (HotStar High Fidelity Polymerase kit, Qiagen) were performed on the purified genomic DNA to produce products for cloning and for T7 assays. For cloning purposes, primers pBSNY5For (GGCATGCTCGAGCTCAGATGCTGCATAT) and pBSNY5Rev (CATGCCTCTAGAAGTGGGTTCCCTAGC) were used with the genomic DNA to produce a 114 bp insert for the XhoI/XbaI digested pBlueScript II SK(-) vector. Clones produced were sequenced with M13Reverse primer.

### Construction of mutated HIV proviral plasmid

To engineer a mutant HIV-1 proviral DNA based on a sequence identified as a genomic edit induced by TALEN cleavage, overlap extension of two PCR products was performed, followed by a PCR using a forward primer (pLAI.28For) and a reverse primer (pLAI.2888Rev). PCR product 1 (7–553) was generated using plai.2 (a full-length HIV proviral DNA) as a template, pLAI.28For and a mutagenic reverse primer (pLai.2Mut1Rev) containing a deletion of 13 nucleotides (positions 531 to 543). PCR product 2 (517–888) was generated using plai.2 as a template, a mutagenic forward primer (pLai.2Mut1For), and plai.2888Rev. The generated insert was ligated into XbaI/ClaI digested plai.2. The mutated region contained within the full length HIV proviral DNA plasmid was confirmed via DNA sequencing.

## Results

### Selection of TALEN target sites and design of TALENs

**Fig 1:**
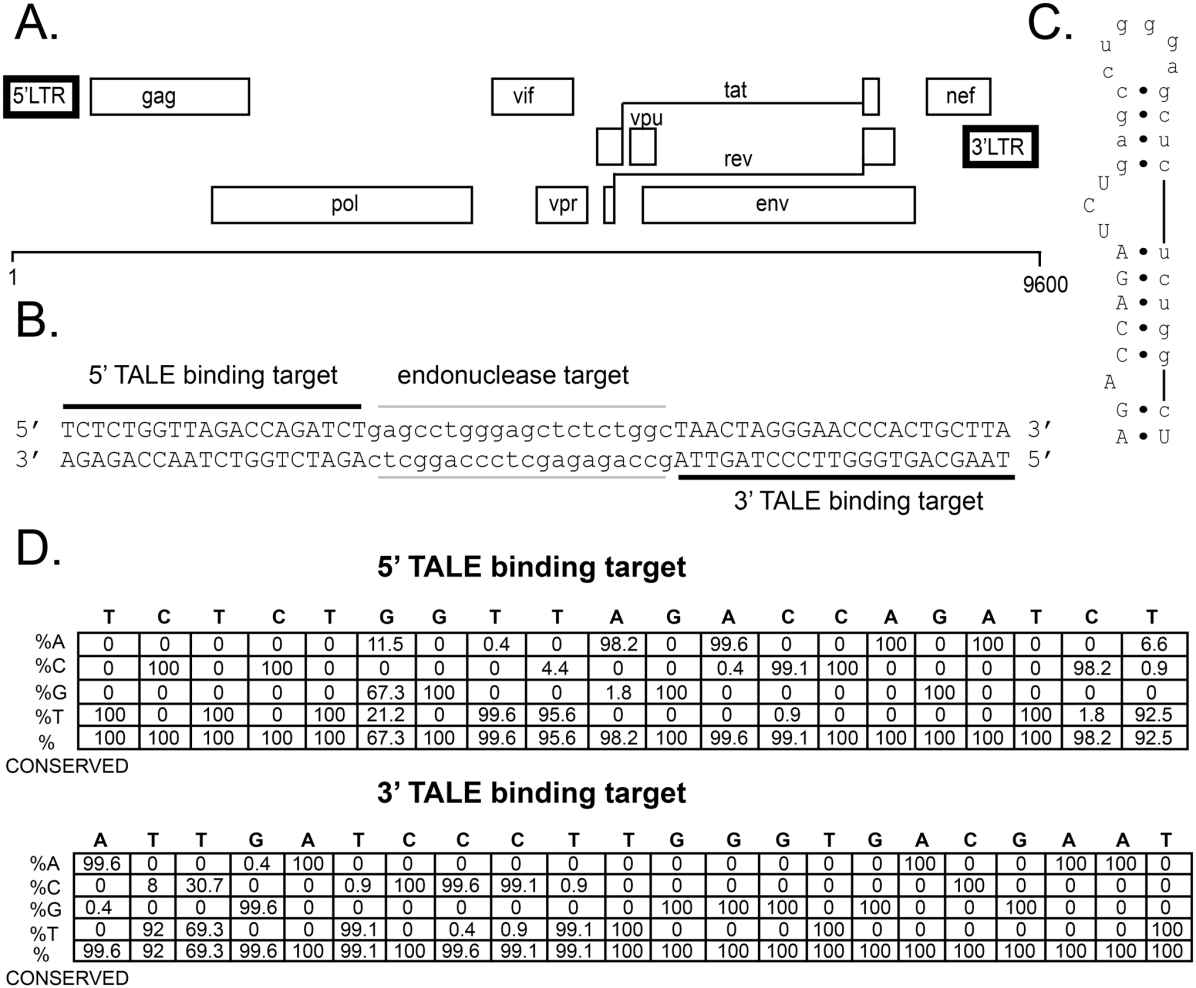
HIV-1 genome conservation analysis to select TALEN sites. A. Schematic diagram of HIV-1 genome adapted from the Los Alamos National Laboratory HIV website [44]. Bolded boxes are regions with HT-TALEN DNA targets, one of which is shown in B. B. 5’ LTR DNA TALEN target sequence. The TALE binding targets are indicated by black lines. The endonuclease target site sequence is in lower case font and indicated by grey lines. C. TAR RNA with partial 5’ TALE binding site in upper-case font and endonuclease target site in lower-case font. D. HIV-1 DNA sequences (274,874 total) from the Los Alamos HIV Sequence Database were aligned with ClustalΩ to determine conservation which is presented in a position specific-scoring matrix [33,44]. The nucleotide frequency for the most conserved regions were chosen as TALEN target sequences found in the TAR coding region (B) of the LTRs (226 sequences) (A).

The first step in designing TALEN pairs was identifying a highly conserved target region of the HIV-1 proviral DNA genome that would not be as prone to mutation and therefore less likely to produce TALEN resistant HIV strains. HIV-1 subtype B DNA sequences from the Los Alamos HIV sequence database were aligned by region and nucleotide conservation was determined. Of the alignments performed, the HIV Long Terminal Repeat (LTR) region (226 DNA sequences) contained nucleotide stretches of the highest conservation [33,44]. These regions encompassed the trans-activation response element (TAR) of the 5' LTR (Fig 1).

Basic Local Alignment Search Tool (BLAST) analysis searching for these sequences in the GRCh38 assembly showed no identical sequences in the human genome [45]. The most similar positions were a matched stretch of 17/20 nt to an intergenic region in chromosome 13 (NC_000013) for the 5’ HT-TALEN, and the next closest were regions with stretches of 14/20 nt identify, two intergenic and one in the coding region of the Glypican 6 gene. For the 3’ HT-TALEN, the most similar match was a stretch of 16/20 nt, matched to an intergenic region in chromosome 11 (NC_000011) and no other stretches with more than 13/22 nt were observed.

Most positions targeted by these TALENs were completely conserved (Fig 1D) and both sites are also completely conserved in laboratory strain NL4-3, but not in all subtype B strains. Mutations that disrupt the TAR stem, in different regions have been shown to abolish viral production, reflecting the high level of sequence conservation [46]. We selected the highly conserved TAR region because this target should be less likely to mutate and produce viable TALEN-resistant escape mutants. Fortuitously, the TALE binding sites in the 5’ LTR were nearly identical in the 3' LTRs. This enables us to potentially damage each site with the same set of TALENs.

Even though we used a bioinformatic analysis to select highly conserved sites as TALEN targets, in reality no sites in HIV-1 are completely conserved. For positions such as the 6^th^, 9^th^ and 20^th^ positions in the 5’ HT-TALEN binding site, these residues are only 67–95% conserved, whereas the remainder of the TALEN binding site is >98% conserved (Fig 1D). HIV with escape mutations can produce resistance to ARV drugs, which may limit the potential use of GEEN for targeting integrated proviral DNAs derived from reverse transcription. The TALEN gene editing technology has the advantage over other GEEN technologies in that a NS repeat variable di-residue (RVD) variant encodes degenerate nucleotide recognition. This can be used to design custom TALENs that encode predicted potential degenerate positions [14,47]. Thus, TALENs can be engineered to tolerate predicted escape mutants.

**Fig 2:**
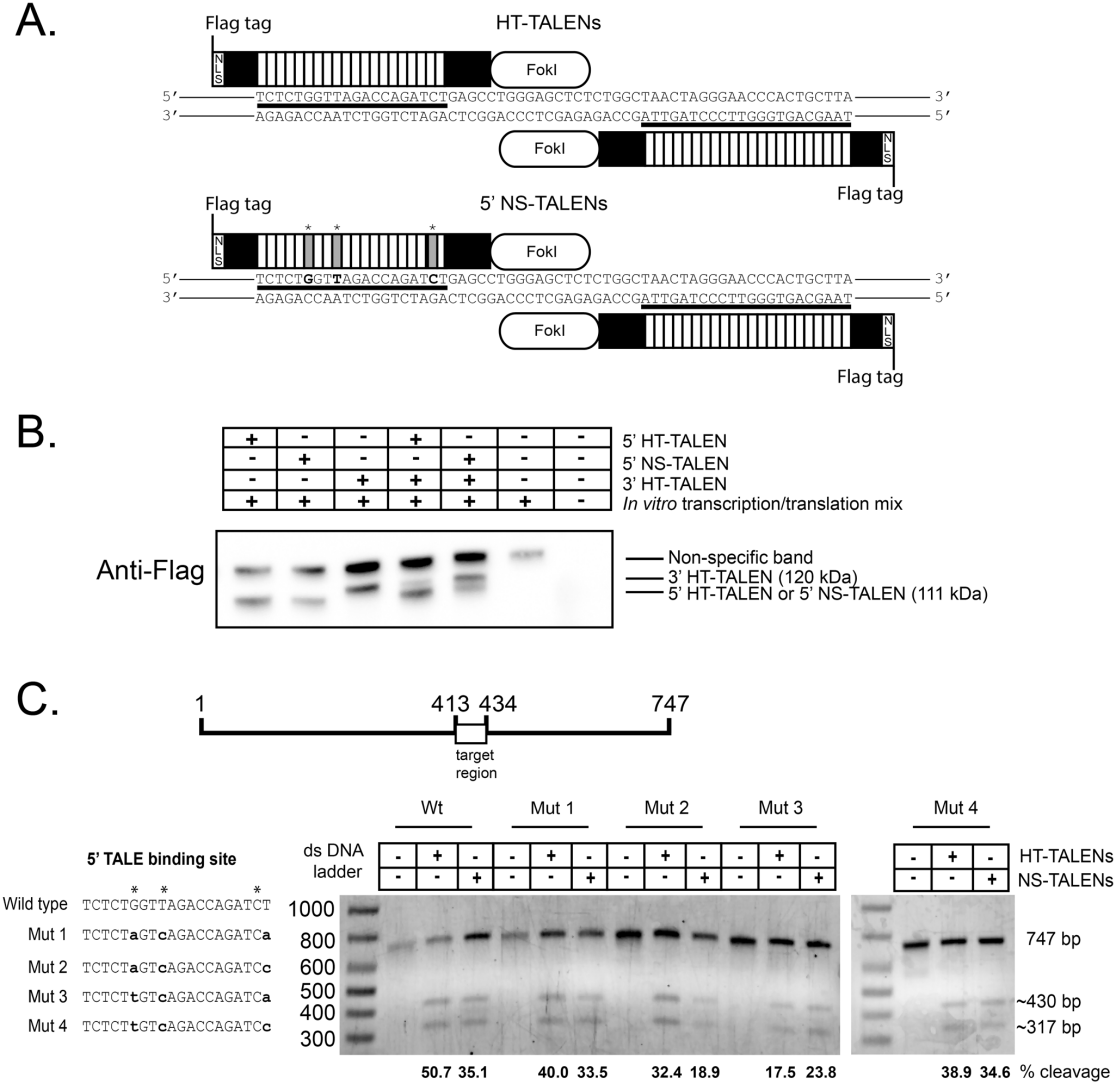
HT-TALENs and NS-TALENs cleave an HIV-1 DNA fragment *in vitro*. A. Schematic diagram representing HT-TALENs and NS-TALENs bound to their cognate DNA target sequence (thick lines). Relative locations of the Fok1 endonuclease, Flag epitope tag, and nuclear localization sequence (NLS) are indicated. Asterisks and grey boxes designate where a “NS” coding TALE repeat was used in the 5’ NS-TALEN construction. B. Western blot of *in vitro* transcription/translation reactions containing no expression plasmids, each TALEN alone, the HT-TALEN pair, or the NS-TALEN pair. C. Gel electrophoresis analysis of *in vitro* cleavage reactions containing no TALEN plasmids, the HT-TALEN pair, or the NS-TALEN pair. The HIV-1 target DNA fragment size is 747 bp, with expected on-target cleavage products of approximately 430 bp and 317 bp. Quantification of cleavage was performed using ImageJ software and is shown below the gel image. D. The HIV-1 target DNA fragment from (C) was mutated in the 5’ TALE binding site to create a set of triple mutant templates (Mut1-Mut4). The sequences of Mut1-Mut4 are depicted in bold, lowercase font and mutated positions are indicated by asterisks. Cleavage reactions containing either the HT-TALEN or NS-TALEN pairs incubated with the HIV-1 target templates were size fractionated by electrophoresis and quantified by densitometry with ImageJ [39].

In addition to the 5’ TALEN that is designed to recognize the canonical 5’ TALE binding site, we also designed another 5’ TALEN construct with NS-TALE monomers positioned to recognize the three more poorly conserved positions in the 5’ TALE binding site. We tested whether or not this is a feasible approach for addressing the degenerate positions (Fig 2A). To differentiate the TALEN pairs in this text, based on the 5’ TALEN recognition sequence, we will designate the pair containing the canonical 5’ TALEN as HT-TALENS and the other pair containing the 5’ NS-TALEN as the NS-TALENs. The NS-TALENs were used to test whether or not it was feasible to cleave the wild type and different triple mutant target templates containing predicted escape mutations.

### TALEN pairs cleave the HIV-1 target DNA *in vitro*

Using the REAL assembly kit, we constructed recombinant plasmids that encoded the 5’ and 3’ HT-TALEN and the 5’ NS-TALEN proteins recognizing the cognate target LTR sequences [48]. The architecture of the repeats and their recognition sequence are shown in Fig 2A. Expression of the Flag epitope-tagged TALENs was verified by *in vitro* transcription/translation reactions and Western blot analysis with a Flag antibody (Fig 2B, S1 File). TALEN protein expression of the expected molecular mass was observed in samples containing the TALEN plasmids, but not in extracts lacking the plasmids. The 3’ HT-TALEN was expressed as a120 kDa protein while the 5’ HT-TALEN and the 5’ NS-TALEN were expressed as 111 kDa proteins. No smaller sized bands were observed, indicating that these proteins are not unstable *in vitro* (S1 File). A higher molecular mass non-specific immunoreactive band was observed in all *in vitro* transcription/translation samples regardless of TALEN plasmid presence.

The endonuclease activity of the TALEN pairs was tested on a 747 base pair HIV-1 proviral DNA PCR product fragment containing the TALEN target sites, as well as HIV-1 proviral DNA PCR product fragments that contained predicted mutations at the 6^th^, 9^th^ and 20^th^ positions of the 5’ TALE binding site (Fig 2C). This DNA was used as a target template to detect TALEN endonuclease activity in cleavage reactions containing the HT-TALEN or the NS-TALEN pair proteins produced by *in vitro* transcription/translation reactions. The HIV-1 DNA target template was cleaved into fragments of the expected sizes when incubated with either TALEN pair, but not when incubated with control extracts lacking the TALEN proteins. We conclude that both TALEN protein pairs cleave the HIV-1 DNA fragment specifically at the target cleavage site. We observed a cleavage efficiency of approximately 42% for both TALEN pairs.

We also tested TALEN DNA target templates containing mutations in the 5’ TALE binding site. Four mutant template with substitutions at three sites (6^th^, 9^th^ and 20^th^) in the 5’ TALE binding site were analyzed. The mutant DNA target templates encoded the 2nd most common nucleotide for each position. We observed that both the HT-TALEN and NS-TALEN pairs cleave all mutant sequences *in vitro* with similar efficiencies (Fig 2C). Cleavage of the mutant templates by the HT-TALENs can be explained by some degenerate recognition by some monomers in HT-TALENs or by the fact that the template and TALEN expression may be higher than that of cells, both of which are addressed in the discussion. Nevertheless, the NS-TALENs can cleave wild type and mutant HIV-1 DNA templates.

### TALEN pairs damage target DNA in live cells

**Fig 3:**
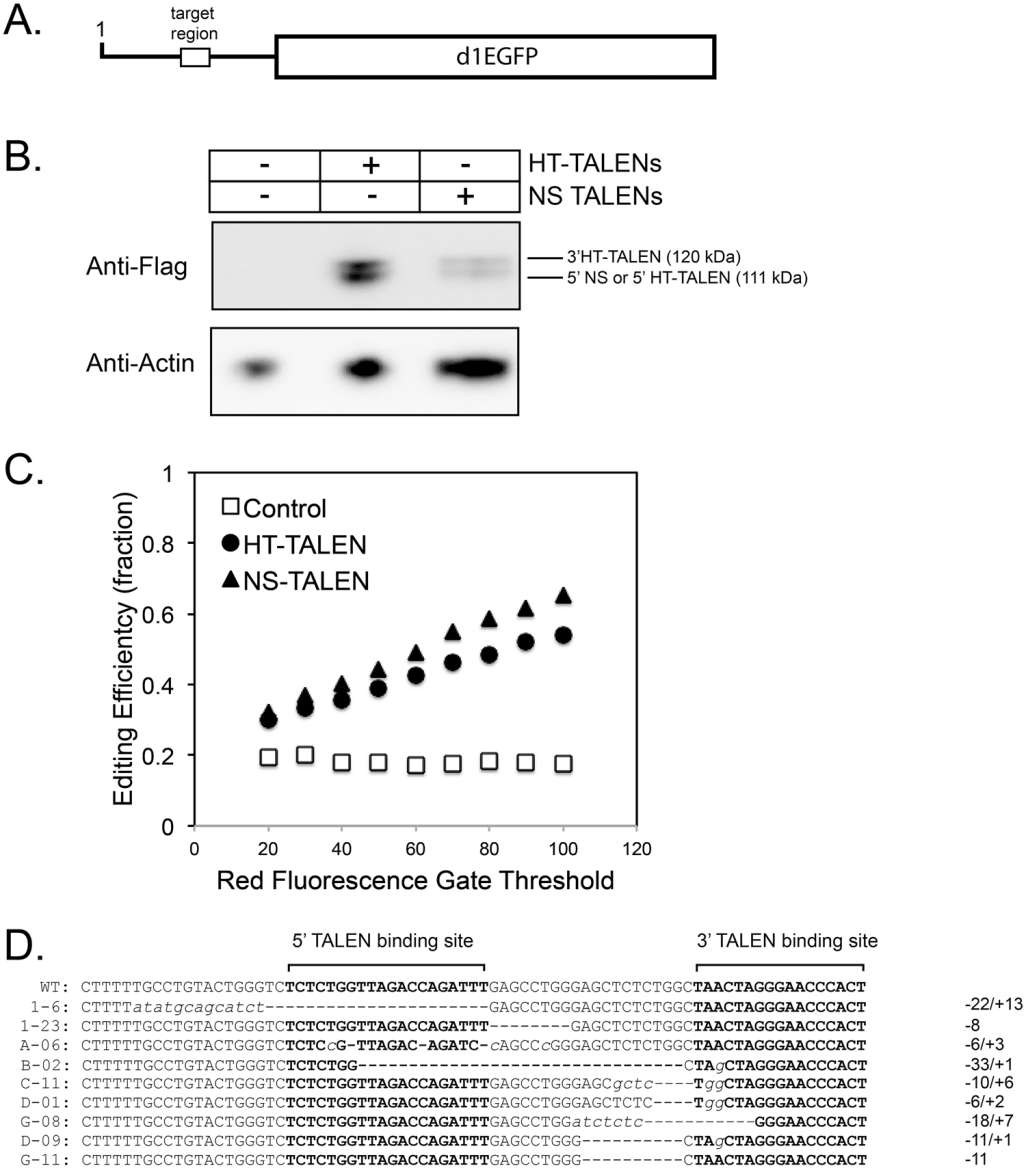
HT-TALEN and NS-TALEN targeting of an HIV-1 LTR in cell culture. A. Schematic diagram of DNA GFP reporter to be targeted by HT-TALENs and NS-TALENs. The target DNA contains the 5’ LTR of HIV-1 fused upstream of the coding region of d1EGFP. B. Western blot analysis of HeLa-tat-III/LTR/d1EGFP cells transfected with either the HT-TALEN pair or NS-TALEN pair. The blot was probed with anti-Flag and anti-Actin as a loading control. C. Dose-response plot based on quantification of flow cytometry analysis of GFP reporter expression. Transiently transfected HeLa-tat-III/LTR/d1EGFP samples were analyzed for GFP and mCherry expression. Cells with mCherry contained the transfected plasmids. Cells containing the functional HIV-1 LTR fused d1EGFP reporter expressed GFP. Samples were done in triplicate. Those samples not expressing GFP, only mCherry were compared. Standard deviations from triplicate samples are smaller than the symbols and not shown. Statistically significant differences between slopes for TALEN treatment and control indicated is by a * (p<0.000001); NS-TALEN and HT-TALENs were not significantly different (p<0.08). D. Sequences of genomic clones containing mutated target regions. Upper-case bolded font indicates designated 5’ TALE and 3’ TALE binding sites. Inserted nucleotides are in lower-case italicized font. A deletion is represented by dashes. Lengths of the insertions (+) and deletions (-) are at the right of each sequence.

We next determined whether the TALEN protein pairs could cleave the TALEN target site in living cultured cells. HeLa-tat-III/LTR/d1EGFP cells stably express a construct containing the HIV-1 5’ LTR (containing the HT-TALEN target site) fused upstream of a d1EGFP coding region (Fig 3A) [40]. GFP is constitutively expressed in these cells and expression is driven by the HIV-1 5’ LTR. These cells were transiently co-transfected with constructs for each TALEN pair and cell lysates were analyzed by Western blot. Expression of the ectopic proteins of the expected molecular masses was observed; however, the NS-TALENs exhibited lower expression (Fig 3B, S2 File).

Our next goal was to test if the TALEN pairs damaged the HIV-1 TAR element in cells using loss of GFP expression as a read out detected by flow cytometry. In addition to non-transfected controls, HeLa-tat-III/LTR/d1EGFP cells were either transfected with pRSET.mCherry alone or pRSET.mCherry co-transfected with constructs for each TALEN pair (S3, S4 and S5 Files). Transfection of either of the TALEN pairs should result in damage to the HIV-1 LTR, thereby reducing GFP expression. We analyzed the transfected cell population that contains pRSET.mCherry using flow cytometry to determine the levels of GFP expression 72 hours post-transfection. A significant difference in the mCherry-only cell populations was dependent on the presence of either TALEN pair compared to the pRSET.mCherry control. The cleavage efficiency is estimated at approximately 30% for both HT-TALEN and NS-TALEN pairs.

We examined if the effect of TALENs on GFP reporter expression was dose-dependent by analyzing the flow cytometry data varying the gating threshold for red fluorescence. The HT-TALEN and NS-TALEN pairs both showed a generally linear dose dependent increase in editing efficiency that was significantly different than control cells (Fig 3C; p < 10^–6^). Although it appeared that the NS-TALENs might have a higher editing efficiency, this was not statistically significant. Notably, these plots did not show saturation of editing efficiency, suggesting that higher TALEN expression would increase editing of the proviral DNA. An editing efficiency of 55–60% was observed for the cells expressing the highest levels of TALEN pairs.

To determine if the targeted region in the LTR contained mutations, the TALEN target region was amplified from DNA isolated from transfected and control non-transfected cells by PCR using primers flanking the target site. Resulting PCR products were subcloned into the pBluescript II SK (-) plasmid and several clones were sequenced. Clones having both deletions and insertions, as well as clones with just deletions were observed. Deletion sizes ranged from 6 to 22 bp in the target region (Fig 3D). Insertion sizes ranged from 1 to 13 bp in the target region. No mutations were observed in 12 sequenced clones of cells transfected with the control pRSET.mCherry vector, while 8 of 29 had mutations for the HT-TALENs, and 2 of 23 were observed for NS-TALENs. This experiment supports the conclusion that HT-TALENs and NS-TALENs can cleave the HIV-1 target DNA site in live cells.

### TALEN pairs damage the integrated complete HIV-1 genome

**Fig 4:**
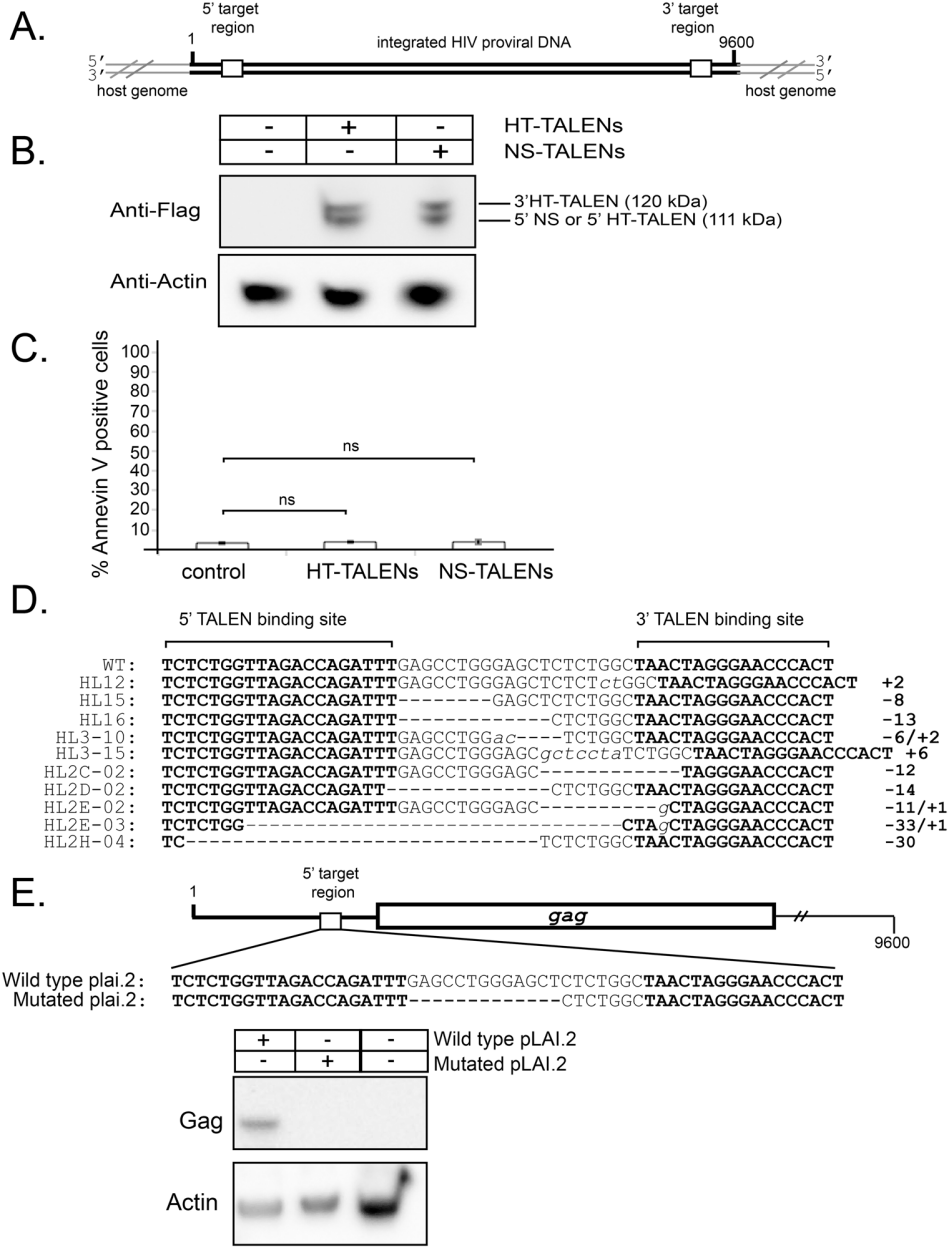
TALEN targeting of integrated complete HIV-1 proviral DNA in cell culture. A. Schematic diagram of the complete HIV-1 proviral DNA to be targeted by the HT-TALEN pair or the NS-TALEN pair. The target region is found in both the 5’ and 3’LTRs. The host genome is indicated in grey. B. Western blot analysis of HeLa/LAV cells transfected with a HT-TALEN plasmid pair or the NS-TALEN pair. The blot was probed with anti-Flag and anti-Actin as a loading control. C. Bar graph showing quantitation of flow cytometry analysis of cytotoxicity. Transiently transfected HeLa/LAV cells were analyzed by flow cytometry (n = 3) to identify Annexin V positive cells. Standard deviations are indicated by error bars with no statistical significance (NS) p>0.05 in cytotoxicity between the control and the TALEN pairs. D. Sequences of clones containing mutated target regions represented as in Fig 3. E. A schematic of the 5’ target region of wild type plai.2 HIV-1 proviral DNA and the mutated plai.2 HIV-1 proviral DNA. The mutated proviral DNA was designed based on the sequence from HeLa/LAV clone HL-16 (Fig 4D). The Gag coding region (containing capsid) is indicated. Western blot analysis of cell lysates harvested from pEAK Rapid cells transfected with mutant or wild type plai.2 proviral DNA. The blot was probed with anti-Capsid qingto detect Gag production and anti-Actin as a loading control.

We determined if the TALEN pairs could edit the full-length integrated HIV-1 proviral DNA in HIV-infected cells. HeLa/LAV cells harbor integrated HIV-1 proviral DNA (Fig 4A) and produce active virus (Fig 4A) [41]. HeLa/LAV cells were separately transiently transfected with either TALEN construct pair and harvested 48 hours post-transfection. Expression of both ectopic TALEN protein pairs was apparent in harvested cell extracts (Fig 4B, S6 File).

We considered that transfection of TALEN constructs may result in cytotoxicity. Therefore, transfection experiments were used to assess cytotoxicity measured by Annexin V staining (Fig 4C, S7, S8 and S9 Files). Triplicate samples analyzed by flow cytometry revealed no significant difference in the number of Annexin V positive cells (p < 0.01) when transfected TALENs were compared to control. We conclude that the TALENs are not significantly cytotoxic to these cells.

Specific editing of the integrated HIV proviral DNA was assessed by amplifying the TALEN target sites from purified genomic DNA, sub-cloning the resulting PCR product into the pBluescript II SK (-) vector, and DNA sequencing of individual clones. Eleven of the 50 sequenced clones contained mutations. Indels were detected with some clones containing both insertions and deletions. Deletion sizes ranged from 6 to 33 bp while insertion sizes ranged from 1 to 6 bp (Fig 4D). This editing profile is typical of that observed in other studies using TALENs, e.g. [20,49,50]. This experiment demonstrates that cleavage by the TALEN pairs induced mutagenesis of the integrated HIV-1 proviral DNA genome. On the basis of these experiments, we conclude that the TALEN pairs can edit integrated HIV-1 proviral DNA in live cells.

The target region in the LTR of HIV-1 is highly conserved and mutations in this region abolish viral production [46]. To assess if mutations resulting from TALEN cleavage of HIV-1 proviral DNA abrogate or limit virus production, a sequence from one of the clones (Fig 4D; HL16) was subcloned into a construct for expression of the full-length HIV-1 proviral DNA. We chose HL16 because it has an indel that deletes the critical stem-loop region of TAR, typical for the majority of other indels we observed. Constructs for the wild type plai.2 and mutant plai.2 HIV-1 full-length proviral DNA were transfected into pEAK Rapid cells (Fig 4E). As an indicator of viral fitness, we examined expression of a key structural virus poly-protein, Gag. Western blot analysis of cell lysates was performed and a Gag band was observed in samples from cells transfected with wild type plai.2 HIV proviral DNA, but not in cells transfected with the mutant HIV-1 plai.2 proviral DNA (Fig 4E, S10 File). Western blot analysis with a loading control antibody to Actin shows similar Actin levels in each sample. This experiment indicates that at least one of the indels introduced by the TALEN pairs can drastically reduce expression of a key viral poly-protein that is necessary for virion production. It is possible that expression is eliminated, but this is not conclusive because of the sensitivity of this assay.

## Discussion

Even with chronic HAART therapy, HIV-1 persists due to latent cell reservoirs containing integrated HIV-1 proviral DNA. These reservoirs can remain inactive for years, not expressing viral proteins or producing infectious virus [3]. Upon activation, previously latent HIV-1 infected memory CD4+ T cells and other cell types, which are not targeted by HAART therapy, can reseed viral infection [51,52]. Upon cessation of HAART therapy, viremia is reestablished in approximately 50 days [53]. In order to eradicate HIV-1 infection, the cells with integrated HIV-1 proviral DNA must be removed or the integrated proviral DNA damaged.

To address viral latency, GEEN technologies are becoming an attractive approach that could be used in combination with HAART therapy [29–31,54]. Tre recombinase and zinc finger nuclease were both previously used to edit an integrated copy of HIV-1 proviral DNA and CRISPR/CAS has been used to remove a GFP reporter flanked by the HIV-1 LTRs [9,12,55]. TALENs have not been used to target the HIV proviral DNA, but were previously used to target the episomal HBV and can reduce viremia in cells and animal models [56].

In this paper, we have used the TALEN technology to target the HIV-1 LTR *in vitro* and in living cells. We demonstrate that HT-TALENs can cleave a HIV DNA fragment *in vitro* and that the HIV-1 LTR is edited in living cells with a ~30% efficiency and a 55% efficiency for cells expressing high levels of HT-TALENs. Furthermore, we show that NS-TALEN variants can recognize wild type and triple mutant sequences providing a strategy for using GEEN to tolerate potential escape mutations.

### Target selection

Recent reviews discussing the use of a GEEN strategy to target the HIV proviral DNA have suggested targeting the coding region of HIV [29,31]. In considering the possibility of escape mutations, we and others performed bioinformatic analyses to select the region of HIV-1 with the highest conserved nucleotide stretches [11,12]. The region with the highest conservation encompassed the TAR region in the LTR [11,12]. One concern with targeting this region was that it might not be accessible due to histone and DNA modification, and DNA packaging. However, we did observe TALEN mediated editing of the TAR site. Improved TALEN delivery systems may increase TALEN editing efficiency in individual cells, resulting in both TALEN target sites in the 5’ and 3’ HIV proviral LTRs being cleaved. This in turn could potentially result in the deletion of the majority of the ~9.6 kb HIV-1 proviral DNA. Large deletions of up to 18 kb have previously been observed with TALENs targeting two local genomic sites [15,57,58]. We did not assay for this deletion because the HIV-1 proviral insertion site in HeLa/LAV cells is currently unknown.

While the TALEN target in the TAR region is not known to be methylated, two CpG islands flanking the transcription start site are close and could affect TALEN binding and cleavage of latent HIV-1 proviral DNA [59]. One of the advantages of using TALENs is that new tools are rapidly becoming available. If methylation is an issue, TALEN variants have been developed to bind methylated cytosines. These TALENs contain RVD regions mutated from “NX” to “N”, which allows recognition of 5-methylated cytosine [60].

### Types of DNA repair

The repair of genome editing technologies is thought to occur by low fidelity non-homologous end joining (NHEJ). In editing of the HIV-1 LTR, we observed small insertions, short deletions, and deletions with insertions. Since DNA Pol μ or λ, are part of this pathway, these polymerases can generate inserts in a template independent manner [61–64], thus may be responsible for the short inserts we observed (2–6 bp) in three clones; this is an editing signature for classical NHEJ [65]. Short deletions of 6–13 bp were observed and are likely due to the exonuclease activity of either Artemis in the classical NHEJ pathway (C-NHEJ), or Exonuclease 1 in the alternative NHEJ pathway (A-NHEJ). Overexpression of Exonuclease I was recently shown to increase TALEN-induced mutation efficiency 30%, suggesting that both NHEJ pathways may be involved in editing of TALEN induced double strand breaks. Cells using only the A-NHEJ pathway (generated by XRCC4 or Ku80 nulls that block the C-NHEJ pathway) typically yield small deletions of 4–25 bp, similar to that we observed with our TALEN pairs [66,67]. The clones having an insertion with deletion are typically observed in other TALEN studies and may represent multiple editing events (e.g.[15,68]). It is noteworthy that improper repair of the targeted TAR region, such as introduction of inserts, deletions, and indels, could negatively affect multiple steps of the viral replication cycle. The 5’ untranslated region (UTR) of the 5’LTR is packed with a variety of RNA regulatory elements with functions that are dependent on proper folding [69,70]. Insertions and deletions, depending on size, could exert severe effects on the ability of the transcribed RNA to achieve necessary secondary structures crucial for transcription.

### Escape mutations

One of the major limitations of treating HIV-1 with ARVs is that drug resistance mutations can arise *de novo* and become transmitted. Since HIV-1 reverse transcriptase is error-prone, mutations could appear in the TALEN binding sites. Based on mutations rates, each HIV-1 proviral DNA is expected to have ~1–3 mutations, thus the mutational landscape in an infected individual is likely to have every possible mutation [71,72]. Polymorphisms in the TALE binding sites may reduce the efficiency of TALEN mutagenesis, thus TALENs could be prone to development of HIV-1 resistance mutations [73]. This is why we used a bioinformatic analysis to select highly conserved sites as targets. Nevertheless, these target sites are not completely conserved, even at the clade level. One strategy we explored was introducing NS RVDs in TALE repeats that enable degenerate recognition of all nucleotides at these select variable target site positions [14,47]. This option to encode degenerate recognition is not yet available in other GEEN technologies, and thus is an advantage of using TALENs.

We built a 5’ NS-TALEN construct having three monomers containing NS RVDs for the positions with less than 98% sequence conservation (Fig 1D). Testing this construct *in vitro* showed that like the HT-TALENs, the NS-TALENs also cleaved the canonical target template. The NS-TALENs also cleaved the four mutant templates tested as expected. However, we were surprised that the HT-TALENs also cleaved our mutant templates. There are two likely explanations for this: (1) the components are more concentrated in this *in vitro* cleavage assay than in living cells, thus driving saturation of the binding site with non-optimal recognition; or (2) that the HT-TALENs have some degenerate recognition.

We favor both explanations. The HT-TALENs encode some degenerate recognition. This may be because the NG repeat variable domains (RVD) in HT- TALENs designed to bind T nucleotides also bind C nucleotides [14]. All of our mutant templates have a T/C substitution in the 9^th^ position and Mut2 and Mut4 have T/C substitution in the 20^th^ position. Thus, this recognition could explain why we observe cleavage of mutant templates by the HT-TALENs. In hindsight, we recognize that this degeneracy should be taken into account in genome analysis to design TALENs to avoid erroneous off-target site cleavage. We suggest that in selected TALEN targeting sites, that this degeneracy is used a priori to BLAST the genome for potential off-target sites. This was not a problem in our application, but could be misconstrued as nonspecific off-target cleaves.

Based on the ability of the NS-TALEN pair to cleave integrated HIV-1 proviral DNA in cell culture, we suggest that this may be an approach to address non-conserved target site positions in integrated HIV-1 proviral DNA. Furthermore, this provides a strategy to address TALEN-resistant HIV-1 strains that may arise by selection, which is important for future use of GEEN technologies in a therapeutic application. Our flow cytometry experiments suggest that the inclusion of NS monomers does not increase cytotoxicity. However, there is delicate balance where introduction of degeneracies could lead to off-target editing in other sites of the human genome, thus this trade-off will need to be evaluated at a more stringent level in future experimentation.

Off-target sites present major potential hazards for GEEN. This can result in mutation of important genes such as tumor suppressors, and induction of cytotoxic responses. The Ousterout et al. study examined cells treated with TALENs targeting the dystrophin gene and detected no off-target edited sites in the exome, as determined by next-generation sequencing [25]. This is supported by another study finding little off-target editing in the exome when targeting 15 different loci [26]. The HIV-1 sequence targeted by our TALEN pairs was selected for its dissimilarities with human genome sequences. Further experimentation will be required to address off-target editing and whether TALENs are the best technology to target integrated HIV-1 proviral DNA.

### Potential for HIV-1 therapy with TALENs

Further experimentation will be necessary before TALENs can be considered for therapy. The next three major steps for moving HT-TALENs toward testing in humans are to increase the efficiency of editing, develop a better delivery vehicle, and test safety and efficacy in HIV-infection animal models. We did observe an overall editing efficiency of ~30% and higher efficiency of 55–60% for the cells expressing high levels of HT-TALENs and NS-TALENs. There have been some major advancements since the original TALENs were constructed at the beginning of these experiments: (1) non-RVD Platinum TALENs; (2) SunnyTALENs; (3) GoldyTALENs; and (4) several versions of obligate Fok1 heterodimers, Fok1 Sharkey mutations, and variable N- and C-terminal TALE repeat lengths [16,18,58,74–78]. Combinations of these HT-TALEN variants are expected to drastically enhance editing efficiency. Furthermore, moving the genes under control of a viral promoter to drive high expression is likely to increase editing efficiency as our dose-response plot showed linearity and no saturation, even at 60% editing efficiency, suggesting that higher TALEN expression will result in more editing.

Improved TALEN delivery systems may increase TALEN editing efficiency in individual cells, and is necessary for *in vivo* delivery. One of the most promising approaches is recombinant high-capacity adenoviral vectors with bicistronic constructs encoding TALEN pairs. Adenoviruses have been previously successfully used for TALEN delivery [79]. Adeno-associated viruses (AAVs), while already in clinical use for lipoprotein lipase deficiency, are too small to deliver TALENs. Experiments with lentiviral vector delivery are prone to TALE monomer rearrangement and have not yet produced targeted editing [79–82]. Other options for TALEN delivery are baculovirus and cell-penetrating peptides, but stability of these proteins in blood is a likely barrier for the latter [83,84].

## Supporting Information

S1 File Expression of TALENs *in vitro*. The western blot from *in vitro* transcription/translation reactions in Fig 2B showing the full gel.
(TIFF)

S2 File Expression of TALENs in HeLa-tat-III/LTR/d1EGFP cells. The western blot of extracts from transiently transfected HeLa-tat-III/LTR/d1EGFP cells in Fig 3B showing the full gel. The blot was probed with anti-Flag.
(TIFF)

S3 File Flow cytometry analysis of pRSET.mCherry transfected Hela-tat-III/LTR/d1EGFP cells. Flow cytometry analysis of GFP reporter expression analyzed to create Fig 3C. HeLa-tat-III/LTR/d1EGFP samples were analyzed for GFP and mCherry expression. Cells containing the functional HIV-1 LTR fused d1EGFP reporter expressed GFP (n = 3).
(TIFF)

S4 File Flow analysis of HT-TALEN transfected HeLa-tat-III/LTR/d1EGFP cells. Flow cytometry analysis of GFP reporter expression analyzed to create Fig 3C. Transiently transfected HeLa-tat-III/LTR/d1EGFP samples were analyzed for GFP and mCherry expression. Cells with mCherry contained the transfected mCherry plasmid and the HT-TALEN pair. Cells containing the functional HIV-1 LTR fused to the d1EGFP reporter expressed GFP (n = 3).
(TIFF)

S5 File Flow analysis of NS-TALEN transfected HeLa-tat-III/LTR/d1EGFP cells. Flow cytometry analysis of GFP reporter expression analyzed to create Fig 3C. Transiently transfected HeLa-tat-III/LTR/d1EGFP samples were analyzed for GFP and mCherry expression. Cells with mCherry contained the transfected mCherry plasmid and the NS-TALEN pair. Cells containing the functional HIV-1 LTR fused to the d1EGFP reporter expressed GFP (n = 3).
(TIFF)

S6 File Expression of TALENs in HeLa/LAV cells. The western blot from HeLa/LAV cells transfected with either the HT-TALEN pair or NS-TALEN pair in Fig 4B showing the full gel. The blot was probed with anti-Flag.
(TIFF)

S7 File Flow cytometry analysis of pRSET.mCherry transfected HeLa/LAV cells following Annexin V staining. Flow cytometry analysis of HeLa/LAV cells transiently transfected with pRSET.mcherry and immmunostained with an Annexin V antibody (GFP channel) to create Fig 4C (n = 3).
(TIFF)

S8 File Flow cytometry analysis of HT-TALEN transfected Hela/LAV cells following Annexin V staining. Flow cytometry analysis of HeLa/LAV cells transiently transfected with HT-TALENs and immmunostained with an Annexin V antibody (GFP channel) to create Fig 4C (n = 3).
(TIFF)

S9 File Flow cytometry analysis of NS-TALEN transfected HeLa/LAV cells following Annexin V staining. Flow cytometry analysis of HeLa/LAV cells transiently transfected with NS-TALENs and immmunostained with an Annexin V antibody (GFP channel) to create Fig 4C (n = 3).
(TIFF)

S10 File Expression of Gag and Actin in transiently transfected pEAK Rapid cells. The western blot from pEAK Rapid cells transfected with either mutant or wild type plai.2 proviral DNA in Fig 4E showing the full gel. The blot was probed with anti-Capsid to detect Gag production.
(TIFF)
