# Supplementary figures and images for: Damaging the Integrated HIV Proviral DNA with TALENs
Source: PLoS One. 2015 May 6;10(5):e0125652. doi: 10.1371/journal.pone.0125652 (PMC4422436; doi:10.1371/journal.pone.0125652)

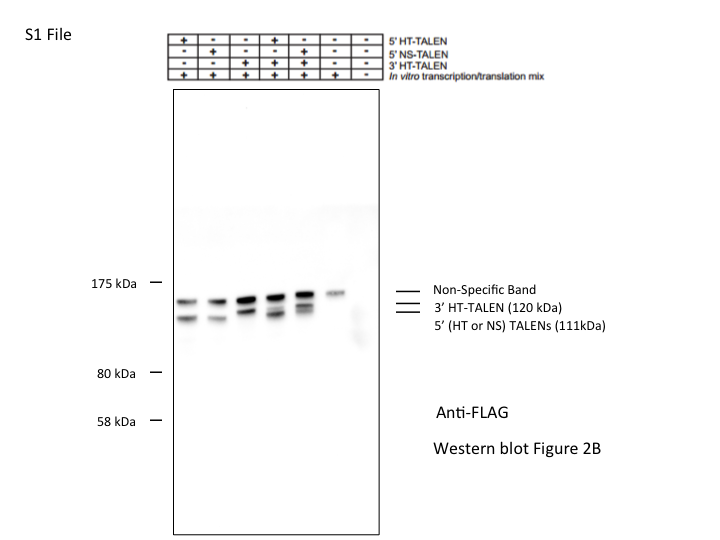

Supplement: S1 File — The western blot from in vitro transcription/translation reactions in Fig 2B showing the full gel. (TIFF) [file pone.0125652.s001.tiff]

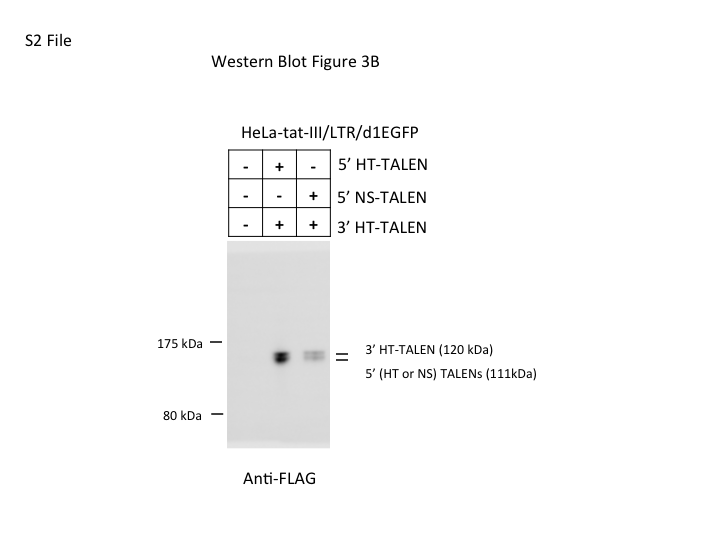

Supplement: S2 File — The western blot of extracts from transiently transfected HeLa-tat-III/LTR/d1EGFP cells in Fig 3B showing the full gel. The blot was probed with anti-Flag. (TIFF) [file pone.0125652.s002.tiff]

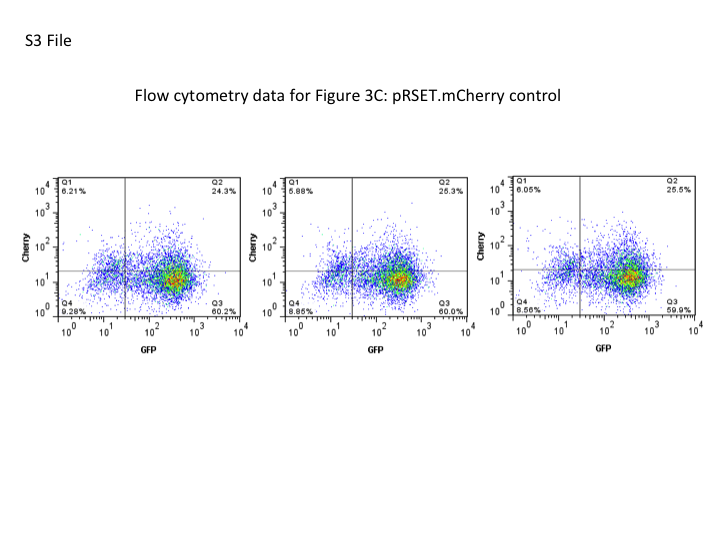

Supplement: S3 File — Flow cytometry analysis of GFP reporter expression analyzed to create Fig 3C. HeLa-tat-III/LTR/d1EGFP samples were analyzed for GFP and mCherry expression. Cells containing the functional HIV-1 LTR fused d1EGFP reporter expressed GFP (n = 3). (TIFF) [file pone.0125652.s003.tiff]

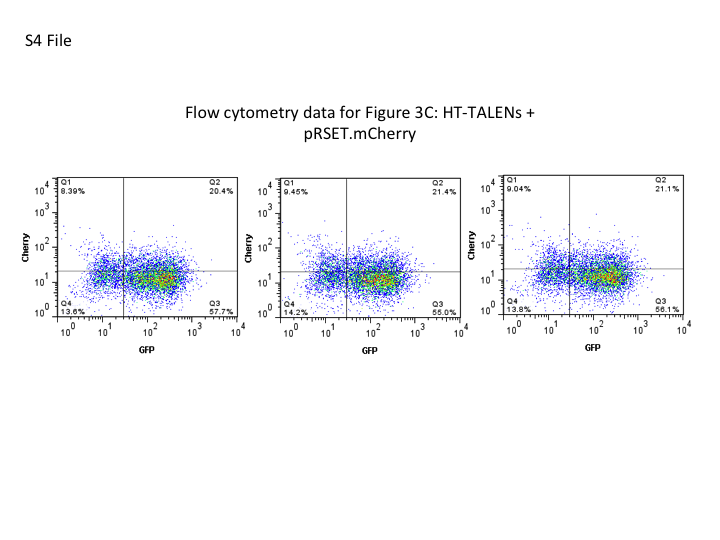

Supplement: S4 File — Flow cytometry analysis of GFP reporter expression analyzed to create Fig 3C. Transiently transfected HeLa-tat-III/LTR/d1EGFP samples were analyzed for GFP and mCherry expression. Cells with mCherry contained the transfected mCherry plasmid and the HT-TALEN pair. Cells containing the functional HIV-1 LTR fused to the d1EGFP reporter expressed GFP (n = 3). (TIFF) [file pone.0125652.s004.tiff]

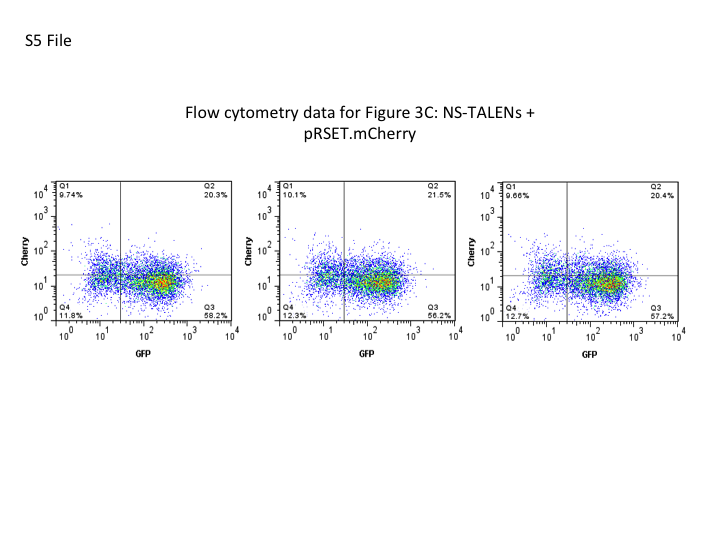

Supplement: S5 File — Flow cytometry analysis of GFP reporter expression analyzed to create Fig 3C. Transiently transfected HeLa-tat-III/LTR/d1EGFP samples were analyzed for GFP and mCherry expression. Cells with mCherry contained the transfected mCherry plasmid and the NS-TALEN pair. Cells containing the functional HIV-1 LTR fused to the d1EGFP reporter expressed GFP (n = 3). (TIFF) [file pone.0125652.s005.tiff]

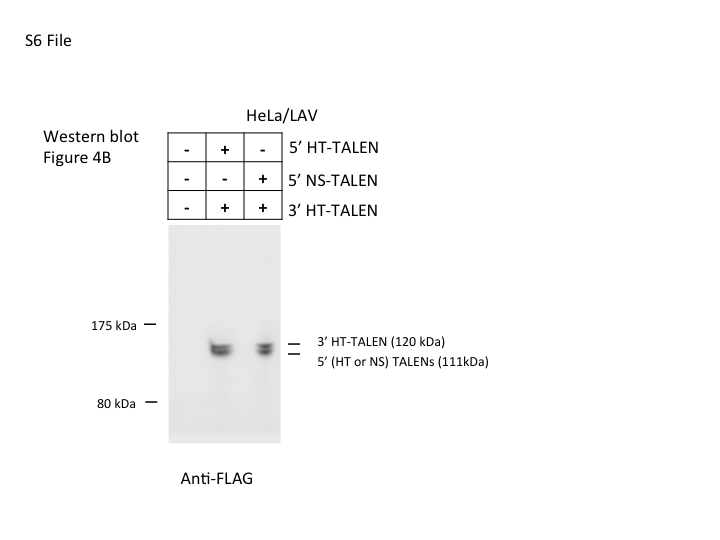

Supplement: S6 File — The western blot from HeLa/LAV cells transfected with either the HT-TALEN pair or NS-TALEN pair in Fig 4B showing the full gel. The blot was probed with anti-Flag. (TIFF) [file pone.0125652.s006.tiff]

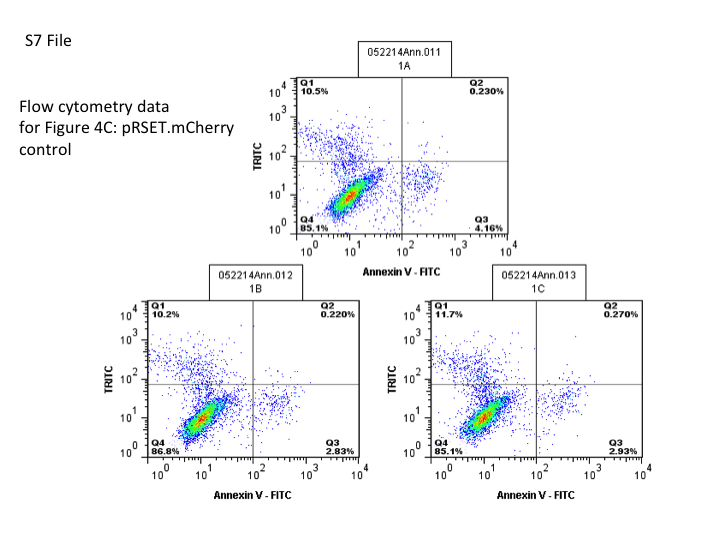

Supplement: S7 File — Flow cytometry analysis of HeLa/LAV cells transiently transfected with pRSET.mcherry and immmunostained with an Annexin V antibody (GFP channel) to create Fig 4C (n = 3). (TIFF) [file pone.0125652.s007.tiff]

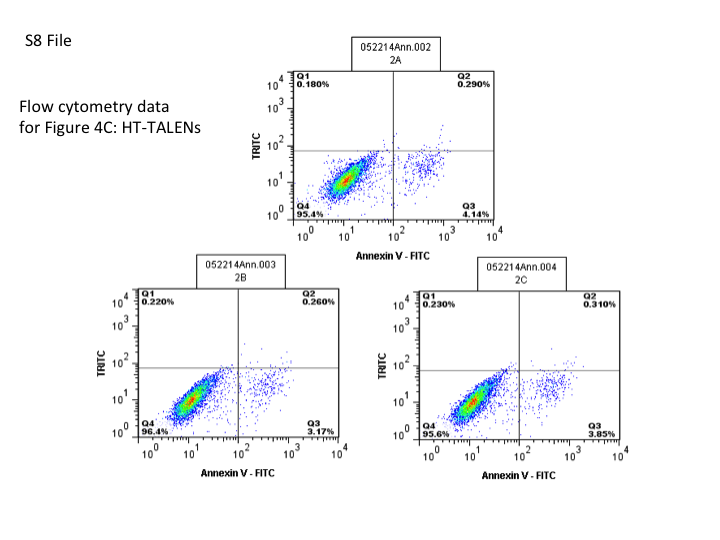

Supplement: S8 File — Flow cytometry analysis of HeLa/LAV cells transiently transfected with HT-TALENs and immmunostained with an Annexin V antibody (GFP channel) to create Fig 4C (n = 3). (TIFF) [file pone.0125652.s008.tiff]

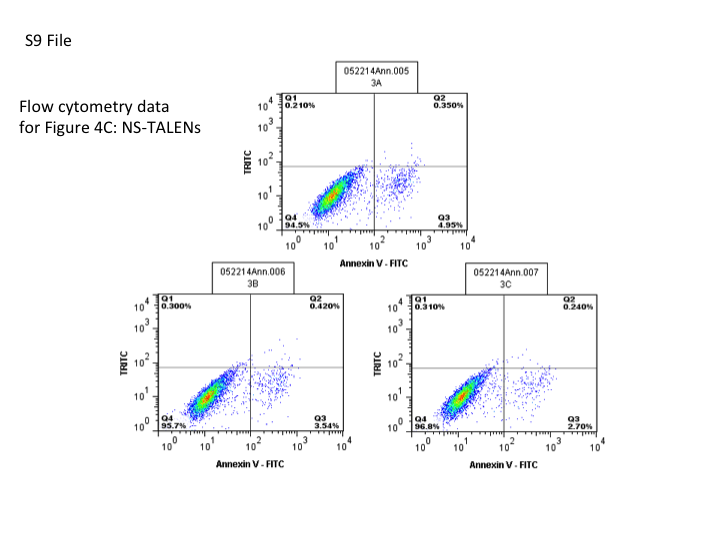

Supplement: S9 File — Flow cytometry analysis of HeLa/LAV cells transiently transfected with NS-TALENs and immmunostained with an Annexin V antibody (GFP channel) to create Fig 4C (n = 3). (TIFF) [file pone.0125652.s009.tiff]

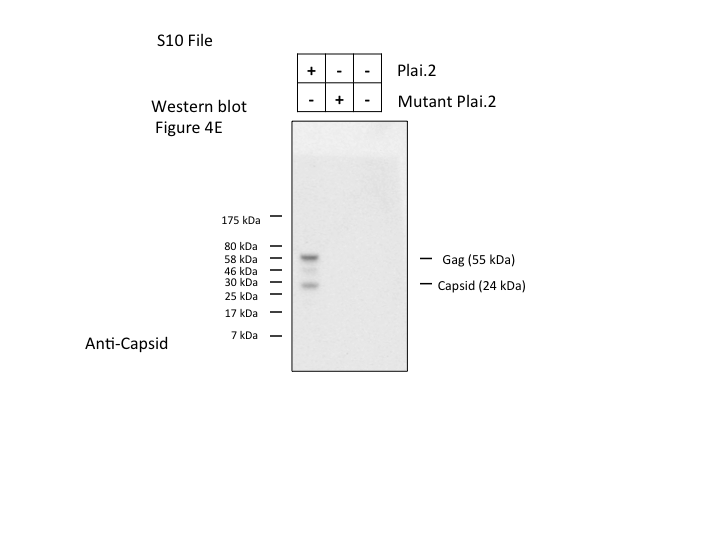

Supplement: S10 File — The western blot from pEAK Rapid cells transfected with either mutant or wild type plai.2 proviral DNA in Fig 4E showing the full gel. The blot was probed with anti-Capsid to detect Gag production. (TIFF) [file pone.0125652.s010.tiff]
